# Research on a Nonwoven Fabric Made from Multi-Block Biodegradable Copolymer Based on l-Lactide, Glycolide, and Trimethylene Carbonate with Shape Memory

**DOI:** 10.3390/molecules22081325

**Published:** 2017-08-10

**Authors:** Joanna Walczak, Michał Chrzanowski, Izabella Krucińska

**Affiliations:** Department of Material and Commodity Sciences and Textile Metrology, Lodz University of Technology, Lodz 90-924, Poland; nonwovens@p.lodz.pl

**Keywords:** shape memory polymers (SMPs), multi-block polylactide copolymer, terpolymer, melt-blown, smart nonwoven fabric, scaffold

## Abstract

The presented paper concerns scientific research on processing a poly(lactide-*co*-glycolide-*co*-trimethylene carbonate) copolymer (PLLAGLTMC) with thermally induced shape memory and a transition temperature around human body temperature. The material in the literature called terpolymer was used to produce smart, nonwoven fabric with the melt blowing technique. Bioresorbable and biocompatible terpolymers with shape memory have been investigated for its medical applications, such as cardiovascular stents. There are several research studies on shape memory in polymers, but this phenomenon has not been widely studied in textile products made from shape memory polymers (SMPs). The current research aims to explore the characteristics of the PLLAGLTMC nonwoven fabric in detail and the mechanism of its shape memory behavior. In this study, the nonwoven fabric was subjected to thermo-mechanical, morphological, and shape memory analysis. The thermo-mechanical and structural properties were investigated by means of differential scanning calorimetry, dynamic mechanical analysis, scanning electron microscopic examination, and mercury porosimetry measurements. Eventually, the gathered results confirmed that the nonwoven fabric possessed characteristics that classified it as a smart material with potential applications in medicine.

## 1. Introduction

A growing number of publications and diverse uses of polymeric-based smart materials demonstrate substantial scientific potential in the engineering and medical field of science [1,2,3,4,5]. The object of this paper is the use of a shape memory polymer based on l-lactide, glycolide and 1,3-trimethylene carbonate in smart textiles. Shape memory materials, classified as intelligent materials have the ability to change their shape or size under certain external stimuli such as temperature, pH, UV, or magnetic and electrical fields [6]. Shape memory polymers (SMPs), in comparison to widely researched shape memory alloys (SMAs), have easier shaping properties, high shape stability and shape recovery, and adjustable transition temperatures [7,8]. The material in the literature called terpolymer is defined as a polymer that consists of three distinct monomers (also known as ABC triblock copolymers). The poly(lactide-*co*-glycolide-*co*-trimethylene carbonate) copolymer (PLLAGLTMC) was obtained in a ring open polymerization (ROP) using zirconium (IV) acetylacetonate as a terpolymerization initiator. The synthesis was described in several publications [8,9,10,11,12,13]. The used zirconium complex was shown to be effective and have low toxicity [12,14,15]. The low toxicity of the zirconium complex was demonstrated by biological assays on the synthesized polymers with the use of this initiator [14,15]. Isotactic poly(l-lactide) is a relatively rigid and brittle material with a crystallinity of approximately 37% and a glass transition temperature in the range of 55–80 °C, while poly(1,3-thrimethylene carbonate) has an amorphous, rubbery nature and a glass transition temperature around −19 °C [10]. Poly(lactide-*co*-glycolide) (PLGA) is a well-known copolymer with decreased glass transition temperature and lower crystallinity and biodegradation rates due to the more random structure in the polylactide after glycolide monomer addition [8,9]. Inclusion of aliphatic cyclic carbonate segmented into PLGA was aimed at lowering the polymer glass transition temperature, increasing plasticity of the copolymer and decreasing the biodegradation rate. Changing the size of each segment in the copolymer, their molecular weights and co-monomer sequencing during synthesis allowed optimization of the properties of the resulting copolymer [8,11]. 

The shape memory terpolymer based on l-lactide (LLA), glycolide (GA), and trimethylene carbonate (TMC) has an amorphous nature [16] with a glass transition temperature (T_g_) near human body temperature, which works as the shape transition temperature (T_trans_) [8]. The thermo-mechanical cycle of the shape memory mechanism consists of several steps: polymer processing and cooling resulting in fixed primary shape creation, mechanical deformation above the polymer transition temperature and cooling to a temperature below T_trans_ with maintained mechanical load, mechanical load removal after which the fixed temporary shape is obtained, polymer reheating above the transition temperature and spontaneous return to the primary shape [17,18,19]. In contrast to other shape memory polymers, which possess microstructure chemical cross-links enabling recovery to the original shape [20], in the studied terpolymer microstructure, only physical interactions can be found that allow maintenance of plasticity and some flexibility at the same time [21]. These physical interactions can be exemplary crystalline domains or chain entanglements, which are created during terpolymer deformation and constitute temporary physical cross-links [11]. In the studied thermoplastic terpolymer, the shape memory mechanism relies on changing the conformation entropy from a low state in the deformation and cooling process to a high state after reheating and shape recovery due to the chain rearrangements [21,22]. The shape fixing occurs due to the decrease in the amorphous phase mobility. 

The shape memory polymers including the researched terpolymer may have several applications as implantable material such as self-expandable stents [9,23,24], self-tightening sutures for wound closure [5,25,26], intelligent scaffolds for minimal invasive surgery [27,28], controllable drug–delivery systems [23,29,30,31,32], or smart elastic memory foams [33] for treating aneurysms [7] or endovascular disorders [34]. The shape memory in those applications based on thermal shape recovery allows the introduction of compressed implants through the narrow passages and returning to the original, deployed shape upon the change in temperature inside the body. Apart from the shape memory characteristic, the terpolymers based on PLGA and TMC are biocompatible and bioresorbable polymers, which makes them more valuable materials as medical implants. The biocompatibility and biodegradability were confirmed by authoritative in vitro and in vivo studies [35,36,37,38,39,40,41,42,43,44]. In vitro tests on human chondrocytes settled on the PLLAGATMC matrix confirmed its biocompatibility before and after the degradation process, which lasted 90 days, and there was 7.4% mass loss. [38] In vivo biocompatibility research on a drug-eluting stent matrix from terpolymer implanted in coronary arteries with 115% overstretch tested on a domestic pig model reported complete healing and biocompatibility with a gradual implant mass loss after 90 days from implantation [39]. In the literature, in vitro and in vivo studies in rats have shown that shape memory poly(lactide-*co*-glycolide-*co*-trimethylene carbonate) (PLLAGATMC) may be effectively used as 17-β-estradiol release system in neurological disease treatment [40]. Taking into consideration several research studies on the PLLAGATMC degradation process, it may be concluded that monomer composition, copolymer molecular weight, and the degradation process conditions have a direct influence on the biodegradation rate [41,42].

In the world of discovering new smart materials and their processing technologies, intelligent textiles start to play a significant role in this research area and commercial applications [6,45]. Smart textiles have the ability to interact with the change in environment and respond in a programmed manner [46]. Currently, smart textiles and shape memory polymers are designed for creating garments of special functionality such as controllable breathability and water vapor permeability [6,47], textile-based drug release systems [48] or garments with wrinkle retention properties upon thermal treatment [49]. The SMPs may constitute components of the fabric, such as interspersing fibers with other yarns when they are woven or as a coating or lamination on fabric. Another method is to create the fabric directly from the shape memory polymer in the textile processing techniques. In this paper, melt blowing was used to produce smart nonwoven fabric from poly(lactide-*co*-glycolide-*co*-trimethylene carbonate) terpolymer as a final product for the first time. Nonwoven material produced with the melt blowing technique is characterized by high porosity with microsized pores and randomly distributed monofilaments in the nonwoven fabric structure. In the literature, electrospun fabrics are fabricated from shape memory polymers of similar characteristics to copolymer, which is the object of this paper [50,51]. This example constitutes research on fibrous scaffolds made of poly(d,l-lactide-*co*-trimethylene carbonate) with shape memory made with an electrospinning technique with the intention to apply the material in minimally invasive surgery [52]. The multi-block PLLAGLTMC terpolymer used in this paper for nonwoven fabric production may constitute favorable material as an intelligent self-tightening scaffold, which with the combined stem cell therapy, may turn out to be a promising strategy for exemplary cardiac disease treatment [53,54]. 

Taking all the previous studies on poly(lactide-*co*-glycolide-*co*-trimethylene carbonate) (PLLAGLTMC) terpolymer and its investigated properties into account, it can be considered a novel shape memory fibrous material that can be used as an intelligent scaffold to promote regeneration of functional tissues and organs. In the following research, the morphological, thermal and shape memory properties of the produced material were investigated to evaluate its potential application in the medical field. 

## 2. Results and Discussion 

### 2.1. Thermal Characteristics of the Used Polymer and Fabricated Materials

**Figure 1:**
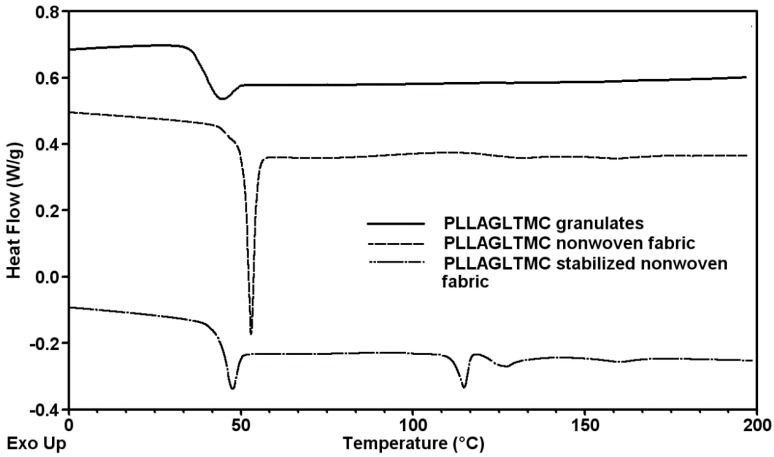
The characteristic temperature determination using differential scanning calorimetry (DSC) analysis recorded during the first heating.

The thermal analysis conducted for differential scanning calorimetry showed the variation in the thermal behavior of the poly(lactide-*co*-glycolide-*co*-trimethylene carbonate) copolymer (PLLAGLTMC) polymer and the nonwoven fabric before and after the thermal stabilization process (Figure 1). As shown in Figure 1, the polymer processed with the melt blowing technique and thermal stabilization process influenced the shape of the glass transition temperature and affected the appearance of the endothermic melting peak. The differential scanning calorimetry (DSC) results showed that the product of the terpolymerization reaction characterized the amorphous, single-phase structure with a glass transition temperature of 42 °C (Table 1). This behavior may have resulted from the granulating process in which the polymer was subjected to high temperatures and then cooled quickly. The effect of rapid cooling was inhibition of the formation of the crystalline phase in the polymer and the formation of amorphous granulates. The amorphous characteristic of the terpolymer led to a Vicat softening temperature of 78 °C. After the polymer processed the thermogram of the changes in heat flow at approximately 131 °C and 159 °C, there were barely noticeable endothermic peaks suggesting the appearance of minor crystallites. What is more, in the stabilized nonwoven fabric, the enthalpy of melting was clearly visibly increased and an additional endothermic peak at 115 °C associated with thermally induced crystallization appeared. A high enthalpy relaxation peak in the nonwoven fabric may result from the fabrication process that was attenuated in the air, as the fibers possess high internal tensile stresses due to the drawing conditions. Furthermore, slightly shifting to a lower glass transition temperature and decreased enthalpy relaxation peak suggested that, during the stabilization process, thermal stress relaxation in the fibers occurred, leading to reduced mobility of macromolecular chains and finally nonwoven fabric shrinkage elimination. The additional onset glass transition temperature, which indicated the beginning of glass transition, was useful for determining the transition temperature in the shape memory evaluation experiments. Taking into consideration the relatively low enthalpy of melting in all studied nonwoven fabrics before and after the thermal stabilization process, material crystallinity was not significant. 

### 2.2. Nonwoven Fabric Shrinkage and Thermal Stabilization Results

**Figure 2:**
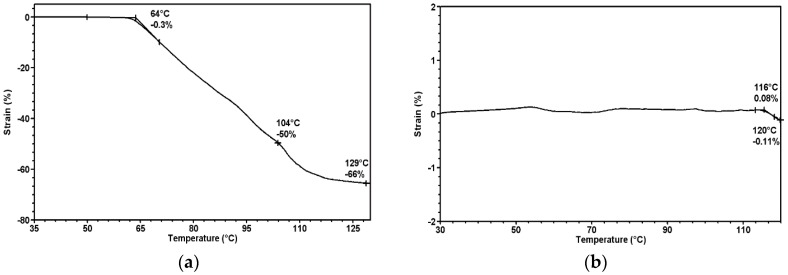
Strain versus temperature dependence measured in a dynamic thermo-mechanical analysis of (**a**)—nonwoven fabric before the thermal stabilization process; (**b**)—nonwoven fabric after the thermal stabilization process.

The characteristics of the melt blowing technique are randomly distributed fibers in a nonwoven structure and a high nonwoven fabric shrinkage ratio at temperatures above T_g_ (Figure 2a). A large change in dimensions during thermal treatment was observed in the dynamic thermo-mechanical analysis (DMTA), where strain versus the temperature curve resulting from high internal stresses in fibers after nonwoven fabric formation. The nonwoven shrinkage started at 64 °C, and the shrinkage ratio reached 66% at 129 °C. As a result of the thermal stabilization process, there was a change in the physical fiber microstructure and the removal of internal stresses in the fibers. The most effective stabilization temperature and time leading to eliminate fiber shrinkage were set empirically to set the final nonwoven fabric shape and dimension. The thermogram in Figure 2b shows that the nonwoven fabric shrinkage was almost eliminated up to 120 °C.

### 2.3. Physical and Structural Characteristics of Terpolymer Nonwoven Fabrics

The nonwoven fabric physical characteristics, such as mass per unit area, thickness, fiber transverse dimensions, total pore area, and average pore diameter, are shown in Table 2.

**Figure 3:**
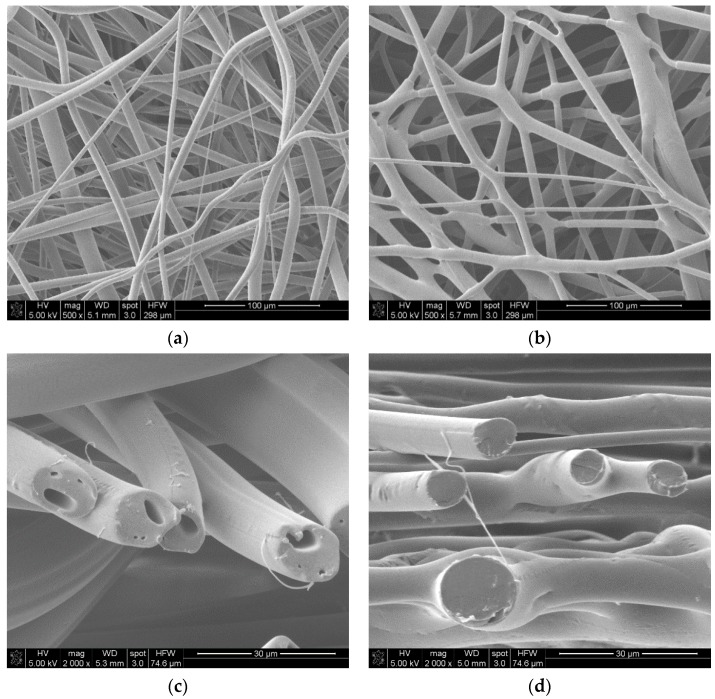
SEM images of (**a**)—nonwoven fabric surface morphology; (**b**)—nonwoven fabric after the thermal stabilization surface morphology; (**c**)—cross-section of the nonwoven fabric; (**d**)—cross-section of the nonwoven fabric after the thermal stabilization process.

Fabricated with the melt blowing technique, nonwovens are characterized as randomly oriented structures with spaces between the fibers (Figure 3a). The nonwoven structure consists of microfibers of minimum transverse dimensions of 0.8 µm and maximum dimensions of 18.6 µm. Figure 3c,d revealed that the fiber cross sections in the nonwoven fabric were regular and close to a circular shape. The diameter of the fibers in the nonwoven fabric were characterized by high coefficients of variation, in particular in the nonwoven fabric after the thermal stabilization process. There was a noticeable decrease in fabric thickness and increase in mass per unit area in the nonwoven fabric after the stabilization process due to the microstructural rearrangement of the fibers in the nonwoven fabric. Densification of the nonwoven fabric microstructure after thermal treatment was clearly visible in the SEM surface morphology examination (Figure 3b) and in the apparent density value (Table 3). What is more, the fibers partially fused to each other due to the temperature treatment, creating a more interconnected and linked structure by physical bonding.

**Figure 4:**
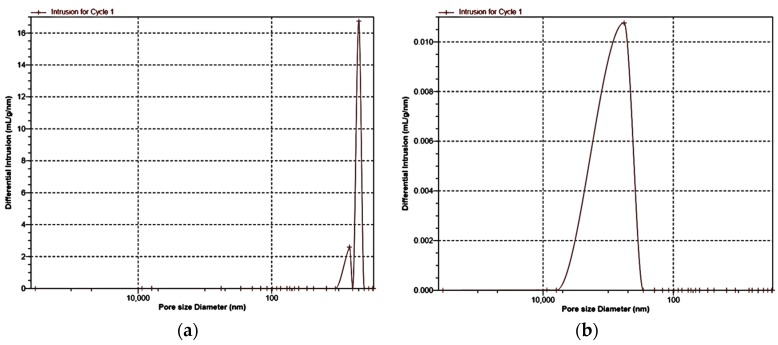
Differential intrusion (pore volume) versus pore size diameter in (**a**)—nonwoven fabric; (**b**)—nonwoven fabric after thermal stabilization.

The changes in the microstructure after thermal stabilization significantly influenced the porosity of the nonwoven fabrics. The mercury intrusion into the nonwoven fabric was much lower in the nonwoven fabric after thermal treatment, which may be explained by its structural transformation and densification. The decrease in the total pore area was related to the increase in the average pore diameter. That relation indicated by the mercury porosimetry analysis results showed spaces between fibers in the nonwoven structure. In Figure 4a, the shape of the curve of differential pore volume versus pore size showed that the dominant pore ranged in size from 4 nm to 6 nm, and from 6 nm do 11 nm in the material. These pores can be associated with the pores within the fibers visible on the cross-section images in Figure 3c. In the nonwoven fabric after the thermal stabilization process, the porosity of the fibers was not visible in the SEM cross-section images (Figure 3d), which can be explained by closing the pores due to the partial fibers melting in the stabilization process. In the nonwoven fabric after thermal treatment, the largest number of pores were detected in the range of 0.6 µm to 9 µm (Figure 4b). The average pore diameter may be associated with spaces between the fibers in the nonwoven fabric after thermal treatment.

### 2.4. Shape Memory Evaluation

**Figure 5:**
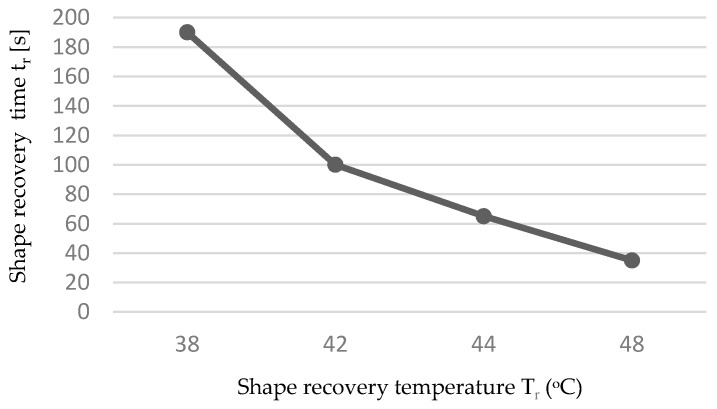
Dependence on the shape recovery time versus shape recovery temperature in the shape memory spiral shaped nonwoven fabric experiment.

**Figure 6:**
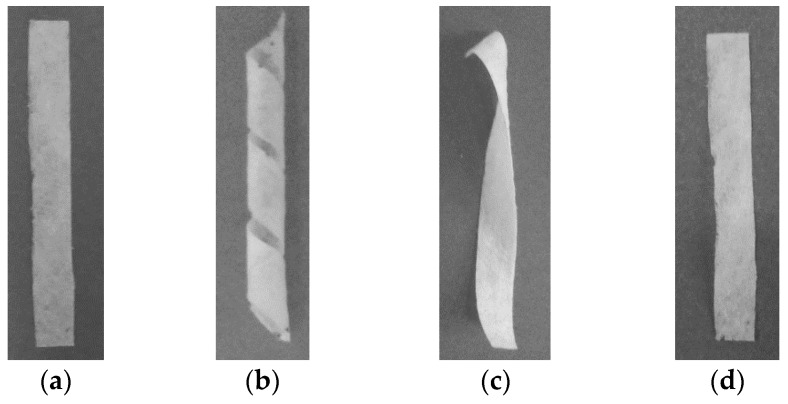
Shape memory evaluation on spiral-shaped stabilized PLLAGLTMC nonwoven fabric, (**a**)—initial shape; (**b**)—temporary fixed spiral shape; (**c**)—shape after recovery at 38 °C and 75 s; (**d**)—shape after recovery at 38 °C and 190 s.

Shape memory was evaluated in the spiral and tensile shaped forms of the fabric produced from the PLLAGLTMC terpolymer and thermally stabilized nonwoven fabrics. The shape memory transition temperature was determined based on the differential scanning calorimetry results. As is reported in the literature concerning research on shape memory PLLAGLTMC terpolymers, the shape recovery activation temperature, T_trans_, was associated with the polymer glass transition temperature, which started below or near T_g_ [11,16]. In the conducted experiments, the shape memory was checked around and above human body temperature. The transition temperature T_trans_ was in the range of 38 °C to 48 °C (Table 3). Increasing the temperature in the shape memory experiment, with the spiral shaped nonwoven fabric, decreased the shape recovery time (Figure 5). The most significant decline in shape recovery time was observable at 42 °C, which was associated with the onset glass transition temperature of the stabilized nonwoven fabric. Figure 6 shows the macroscopic demonstration of the shape memory in spiral shaped nonwoven fabrics, which recovered to the initial, permanent shape in 190 s after immersion in water at 38 °C.

**Figure 7:**
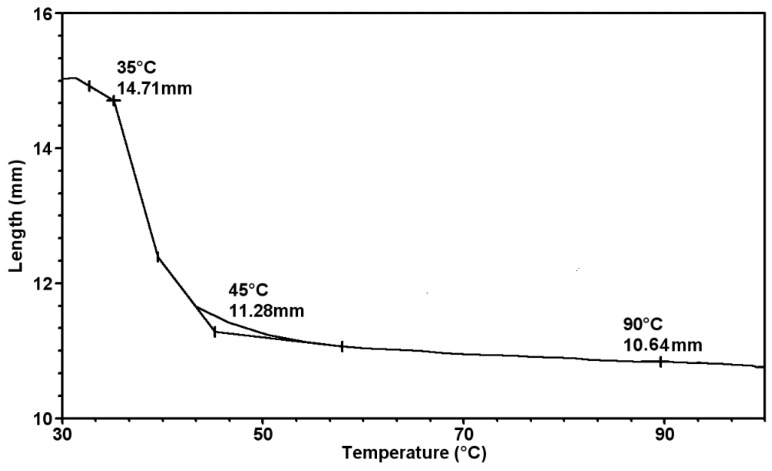
Dependence on the sample length of 100% stretched and stabilized PLLAGLTMC nonwoven fabric versus temperature.

To quantitatively assess the PLLAGLTMC nonwoven fabric shape memory, we conducted a thermomechanical tensile test and calculated the shape recovery ratio according to the methodology described in the methods. All deformed nonwoven fabrics in the tensile test after cooling and stress removal exhibited excellent shape stability at temperatures below 30 °C. Unloading below T_g_ led to no dimensional change in the deformed nonwoven stripes and shape fixity ratio close to 100%. Shape recovery was checked after immersion in water at 48 °C and during fast heating in DMTA analysis. In Figure 7, the sample length versus temperature curve allowed observation of the beginning of shape recovery at 35 °C, and shape recovery did not increase drastically when the temperature reached 45 °C. In the experiment conducted with DMTA apparatus, the maximum shape recovery ratio was 85%. Here, higher shape recovery ratios were obtained after stretched samples were immersed in water at exact temperatures and times. It is also worth noting that more effective shape recovery is observed with higher tensile deformation when comparing the shape recovery of two different tensile samples in water at the same time and temperature. The shape memory in the studied multi-block copolymer depends mostly on chain entanglements during deformation and fixing determined by the mobility of the amorphous phase. After the deformed and fixed sample reheating from a more oriented network, it came back to a more random conformation and shape recovery. The shape memory of the nonwoven fabric depended on several factors such as the type of deformation, programming temperature and time, type of conducted experiment, and sample size, which also turned out to influence shape memory. Apparently, in the small stripes used in the DMTA tensile test, there were not enough node-intermolecular attachments created, which are made by formation of crystalline domains or chain entanglements that results in less-than-full shape recovery.

## 3. Materials and Methods

### 3.1. Material Characterization

The starting material for the nonwoven fabric production was a poly(lactide-*co*-glycolide-*co*-trimethylene carbonate) copolymer (PLLAGLTMC) with thermally induced shape memory with the trade name BIOCOP^®^ delivered by Biomatpol PL in the form of granulates. The polymer was synthesized with ring-opening copolymerization of lactide with other cyclic monomers using zirconium (IV) acetylacetonate Zr (Acac) as a reaction initiator as reported by Dobrzyński in 2002 [12]. The share of l-lactide, glycolide, and 1,3-trimethylene carbonate in the copolymer was determined by nuclear resonance spectrometry (NMR), and equaled 69%, 11%, and 20%, respectively. The obtained copolymer had an average molecular weight of Mn = 35,130 g/mol estimated by gel permeation chromatography (GPC).

### 3.2. Polymer Processing

The multi-block copolymer was processed by melt-blown technique using Mini Lab Haake twin-screw extruder. The method is devoted to the thermoplastic polymers. In this technique, the polymer is melted in the extruder and delivered to the spinning nozzle by constant rotary speed of the screws, where is extruded through the die hole. Due to the high-velocity hot and dry air streams, the droplets of the molten polymer are converted into the fibers. The fibers are collected on the rotating drum equipped with air suction device where randomly distributed fibers creating the web. After the fibers solidification the final nonwoven fabric is produced and collected. The influence on the process effectiveness and final product properties like thickness or mechanical strength, has adjusting the proper processing parameters (Table 4) [55].

### 3.3. Test Methods

The experiment was partially carried out in accordance to the described methodology in another author’s publication on shape memory nonwoven fabric produced with melt blowing technology [56].

#### 3.3.1. Thermal Analysis

The thermal properties of the investigated copolymer and nonwoven fabric were analyzed with differential scanning calorimetry (DSC) measurements using a DSC Q2000 device (TA Instruments, New Castle, DE, USA) according to the ISO standard EN ISO 11357:2009. In the DSC analysis, the material’s characteristic temperatures, the glass transition temperature (T_g_), the melting temperature (T_m_) and the crystallization temperature (T_c_), were determined. The thermal properties of the material were examined in the range from −25 °C to 200 °C at a rate of 10 °C/min under nitrogen atmosphere and a flow rate of 50 mL/min. After the first heating cycle, the samples were immediately cooled to −25 °C and reheated to 200 °C (second heating cycle). The melting temperature (T_m_) was determined as the peak maximum of the endotherm. The enthalpy of melting was calculated as 1 g of the sample, which is a normalized value. The glass transition temperature (T_g_) was determined at the half-height of the heat capacity change in the thermal transition. The glass transition temperature can be further divided into starting and ending transition points called onset and endpoint temperatures, respectively. The glass transition temperature is a midpoint temperature. Due to the amorphous nature of the used copolymer, the Vicat softening point was also determined using dynamic thermo-mechanical analysis DMTA Q800 device (TA Instruments, New Castle, DE, USA). It is the temperature at which the flat-ended needle penetrated the material to the depth of 1 mm. 

#### 3.3.2. Thermal Stabilization Process

**Figure 8:**
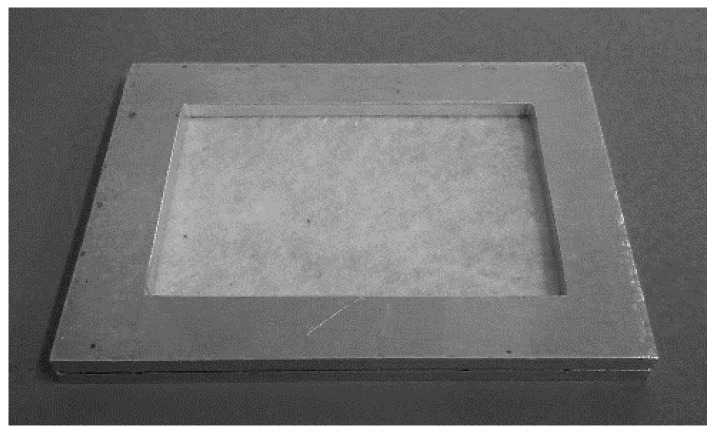
Aluminum frame for the multidirectional thermal stabilization process with PLLAGLTMC nonwoven fabric.

Due to the high shrinkage ratio of the formatted nonwoven fabrics at elevated temperatures, the thermal stabilization process is carried out in specially constructed aluminum frames in an unstressed state. The construction of the tool allowed elimination of the multidirectional internal stresses in the fibers. The nonwoven sheet was placed between the plates and clamped on the sides enabling free microstructure reorganization (Figure 8). The temperature of the stabilization process was 80 °C, and it was carried out for 30 min as determined empirically until the fiber shrinkage was eliminated. After heating the nonwoven fabric, it was immediately cooled in order to freeze the physical microstructure of the fibers and inhibit crystallization. The heating medium was hot air.

The shrinkage ratio of the nonwoven fabric before and after thermal stabilization was determined using DMTA Q800 apparatus. DMTA analysis allowed quantitative investigation of the shrinkage ratio as a function of strain change versus temperature.

#### 3.3.3. Nonwoven Basic Structural Characterization

The average mass per unit area and thickness of the nonwoven fabrics before and after thermal stabilization were determined in accordance with the PN-EN ISO 9073-1:1989 and PN-EN ISO 9073-2:2002 standards, respectively. The size of the samples described in the standards was reduced and adjusted to the low amount of test material. The thickness measurements were made by a thickness gauge with a loading of 0.5 kPa and apparatus accuracy of ±0.01 mm.

#### 3.3.4. Scanning Electron Microscopic Examination

Scanning electron microscopy (SEM) investigations were carried out with a high-resolution NOVA NanoSEM 230 microscope (FEI, Eindhoven, Netherlands)) under high vacuum conditions using an accelerator voltage of 5 kV and magnification of 50× to 15,000×. The samples were coated with a gold layer with the ion sputtering method. Two types of microscopic observations were made: surface morphology and cross-section views of the nonwoven fabrics before and after the thermal stabilization process. The transverse dimensions of the fibers in the nonwoven fabric structure were analyzed using the Lucia G software (version 4.8, Laboratory Imaging Prague, Czech Republic) for image analysis. Sixty different fibers from the SEM surface morphology images were selected from randomly chosen places on the nonwoven sample for transverse dimension examination.

#### 3.3.5. Porous Structure Assessment

The average pore diameter and total pore area of the nonwoven fabric structure before and after the thermal stabilization process was assessed with the mercury porosimetry technique. Measurements were made on AutoPore IV apparatus (Micrometrics, Norcross, GA, USA) at a low-pressure port and a high-pressure port. The range of detection of the apparatus was from 3.0 to 400,000 nm. The curves of the dependence between pore volume and pore size were drawn in order to evaluate pore size distribution in the examined samples.

#### 3.3.6. Characterization of Shape Memory Behavior in DMTA Tensile Test

**Figure 9:**
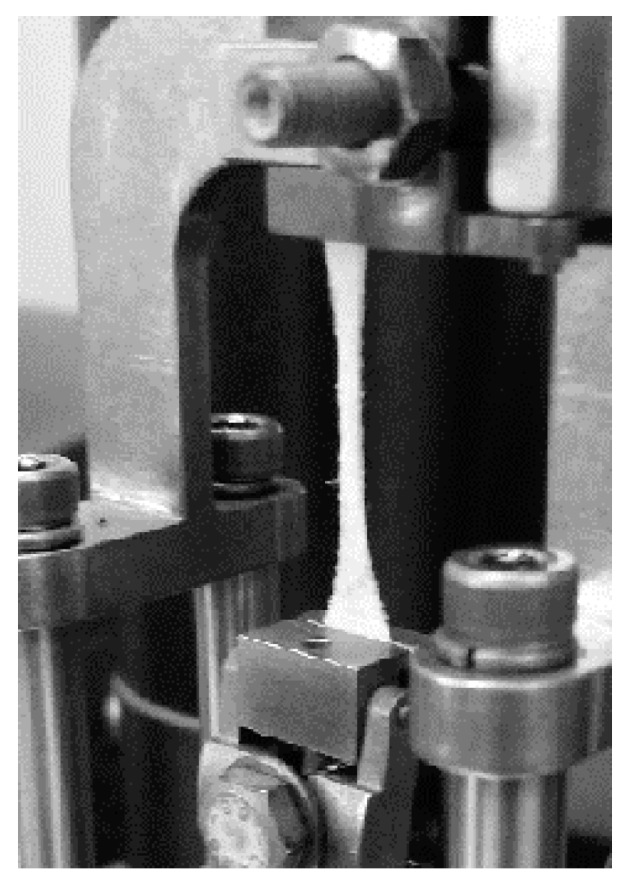
The nonwoven sample temporary shape in the clamps DMA apparatus.

The first experiment aimed to evaluate shape memory in the produced nonwoven fabric and was conducted using thermo-mechanical apparatus (DMTA) in tensile mode. In the first step, the initial shape constituted the 9 mm × 6 mm × 0.5 mm stripe cut from the stabilized nonwoven sheet. The temporary shape was obtained by 50% and 100% of the nonwoven stripe tensile deformation, which occurred slightly above T_g_ at 50 °C in the DMTA clamps (Figure 9).

According to the literature, the deformation temperature should allow easy shaping under the influence of external force, and at the same time, should not facilitate chain disentanglements at higher temperatures that increase chain mobility [11]. The stretching of the sample in the DMTA apparatus occurred with a pretension force of 0.001 N and constant strain rate of 10%/min after the temperature equilibrated. Then, the stretched sample, kept in clamps, was cooled by freeze spray to approximately −67 °C. After the cooling process, the stress was released and the shape was fixed. The shape recovery was examined during nonwoven reheating at a fast rate ≈45 °C/min from 30 °C until it reached equilibrium at 120 °C. Additionally, the shape memory of 100% and 50% stretched samples was checked after immersion in water at 48 °C. 

The shape memory was quantitatively described by the shape recovery ratio, R_T_, and the shape fixity ratio (R_F_), which were calculated from Equations (1) and (2), respectively,

(1) R_T_ (%) = (L_U_ − L_R_)/(L_T_ − L_I_) × 100

(2) R_F_ (%) = (L_U_ − L_I_)/(L_T_ − L_I_) × 100

where L_I_ is the initial sample length, L_T_ is the length of the sample after deformation (temporary shape), L_R_ is the length of the sample after final shape recovery, L_U_ is the sample length after deformation, cooling and stress released, and T is the shape recovery temperature.

#### 3.3.7. Thermo-Mechanical Shape Memory Experiment with Spiral Shaped Fabrics

Second, the thermo-mechanical shape memory test is analogous to the tensile experiment, but the sample size and temporary shape differ. The initial shape was a stripe with 50 mm × 6 mm × 0.5 mm dimensions, which was cut from nonwoven fabric after thermal stabilization. The stripe of nonwoven fabric was folded on a 3.5 mm diameter rod and kept at the end. The samples were thermally equilibrated at a constant temperature of 50 °C for 30 min under constant stress. The stress was released after the cooling procedure in which the spiral shape was fixed. Finally, free-strain nonwoven fabric in the form of spirals was immersed in water at a temperature range from 38 °C to 48 °C where shape recovery occurred in a measured period of time. 

## 4. Conclusions

In the present paper, we demonstrated that shape memory PLLAGLTMC nonwoven fabric can be successfully fabricated with the melt blowing technique for the first time. On the basis of the performed thermal analysis, it can be concluded that the poly(lactide-*co*-glycolide-*co*-trimethylene carbonate) (PLLAGLTMC) terpolymer had a single-phase amorphous structure. After the polymer processing, in the DSC analysis, we observed two small endothermic peaks in the nonwoven fabric before and after thermal stabilization at approximately 130 °C and 160 °C, which most probably correspond to the structure order as a result of the nonwoven formation process. The appearance of one additional endothermic peak at 114 °C in the stabilized nonwoven fabric indicated the formation of the crystalline phase as a result of temperature exposure over a certain period of time. The structural investigations revealed in the nonwoven fabric were highly porous with randomly distributed microfibers. Highly porous materials enhance cell proliferation and ingrowth and can be dedicated for scaffold design and fabrication. The heat treatment in the stabilization process induced microstructural rearrangement and its densification. As was reported, the thermal stabilization process is the most effective when it starts melting the crystalline regions of the fibers in the nonwoven structure; thus, the chosen stabilization temperature was 80 °C and was around the material softening point [57]. As a result of the conducted thermal stabilization process, there were successfully removed internal stresses in the fibers, which promoted fiber shrinkage at the increased temperature. The deformation above T_g_ and fixed below T_g_ of nonwoven fabrics exhibited excellent shape fixity behavior after unloading, and were able to recover to the initial shape fully or to a great extent. Observing the correlation between the glass transition temperature and shape memory experiment results, it can be noted that the shape recovery started at the onset glass transition temperature. In this paper, the results were in line with what the literature has reported, that the shape memory strongly depended on programming a temporary shape and inducing temporary cross-links evoked by chain entanglements [11]. Varying the type of deformation, the shape recovery temperature and shape memory experimental conditions produced a maximum of a 91% shape recovery ratio. However, in case of spiral-shaped nonwoven fabric, there was full shape recovery in water at an initial recovery temperature of 38 °C. The temperature at which the studied nonwoven fabric obtained full recovery was in the neighborhood of human body temperature, and therefore it raises the possibility of applying the PLLAGLTMC nonwoven fabric in biomedical applications as shape memory implants.

## Figures and Tables

**Table 1:** Characteristic temperatures of the studied materials obtained for differential scanning calorimetry analysis.

Material	Heating Cycle	Glass Temperature T_g_, (°C)	Melting Temperature T_m_, (°C)	Enthalpy of Melting ΔH_m_, (J/g)
PLLAGLTMC granulates	1st	Onset: 35.4 Midpoint: 37.7	-	-
	2nd	Onset: 40.2 Midpoint: 42.3	-	-
PLLAGLTMC nonwoven fabric	1st	Onset: 38.0 Midpoint: 47.4	131.0 159.3	0.5 0.5
PLLAGLTMC nonwoven fabric after thermal stabilization	1st	Onset: 42.3 Midpoint: 43.3	114.8 127.0 160.0	2.3 1.2 0.6

**Table 2:** Physical characteristics of the manufactured poly(lactide-*co*-glycolide-*co*-trimethylene carbonate) (PLLAGLTMC) nonwoven fabric before and after thermal stabilization.

Material	Thickness of Fabrics (mm) (cv, %)	Mass per Unit Area (g/m^2^) (cv, %)	Average Fiber Diameter (µm) (cv, %)	Apparent Density (kg/m^3^)	Total Pore Area (m^2^/g)	Average Pore Diameter (nm)
PLLAGLTMC nonwoven fabric	0.63(9.5)	46.4(13.0)	6.7(34.7)	74.2 ± 0.1	19,233.7 ± 0.1	8.1 ± 0.1
PLLAGLTMC nonwoven fabric after thermal stabilization	0.48(10.0)	56.2(16.7)	6.9(46.2)	116.6 ± 0.1	71.8 ± 0.1	3594.7 ± 0.1

**Table 3:** Shape recovery parameters of the stabilized PLLAGLTMC nonwoven fabrics.

Temporary Shape	Shape Recovery Condition	Shape Recovery Temperature T_r_ (°C)	Shape Recovery Time t_r_ (s)	Shape Recovery Ratio R_T_ (%)	Shape Recovery Evaluation
Spiral	Immersion in water	38	75	-	Close to full recovery (Figure 6c)
Spiral	Immersion in water	38	190	-	Full recovery (Figure 6d)
Spiral	Immersion in water	42	100	-	Full recovery
Spiral	Immersion in water	44	65	-	Full recovery
Spiral	Immersion in water	48	35	-	Full recovery
50% stretched	Immersion in water	48	35	72	-
100% stretched	Immersion in water	48	35	91	-
100% stretched	Heating in DMA	35–90	8 36	78 85	-

**Table 4:** Melt blowing processing parameters used for PLLAGLTMC nonwoven fabric formation.

Air Stream Temperature (°C)	Die Temperature (°C)	Extruder Temperature (°C)	Air Flow Rate (m^3^/h)	Twin-Screw Extruder Rotation Velocity (rpm)	Die Collector Distance (cm)
250	240	150	6	70	15
